# Endoscopic intermuscular dissection of a residual rectal neuroendocrine tumor with high-pressure injection and double-clip traction

**DOI:** 10.1055/a-2173-7103

**Published:** 2023-11-07

**Authors:** Marion Schaefer, Jérémie Albouys, Sophie Geyl, Romain Legros, Timothée Wallenhorst, Mathieu Pioche, Jérémie Jacques

**Affiliations:** 1 26920 Hepatogastroenterology, Nancy Regional University Hospital Center, Nancy, France; 2 37925 Hepatogastroenterology, Dupuytren Hospital, Limoges, France; 3 55065 Department of Gastroenterology, Pontchaillou Hospital, Rennes, France; 4 Endoscopy and Gastroenterology, Edouard Herriot Hospital, Lyon, France; 5 BioEM, XLim, UMR 7252, CNRS, Limoges, France

Endoscopic resection, with advanced techniques such as modified endoscopic mucosal resection (EMR) or endoscopic submucosal dissection (ESD), is the first-line treatment for small grade 1 rectal neuroendocrine tumors (NETs), and complete histological resection is mandatory to avoid overtreatment or unnecessary follow-up 1 . Rectal NETs are rare tumors that can be misdiagnosed and consequently inadequately resected with cold snare, hot snare polypectomy, or biopsy forceps, leading to incomplete resection 2 . In such cases, complementary resection using ESD or transanal endoscopic microsurgery can be proposed to achieve complete resection and dispense with further follow-up 3 . Recently, endoscopic intermuscular dissection has been described to increase the deep resection margin for invasive submucosal rectal cancer 4 .

We report the case of a 26-year-old man with previously incomplete resection by conventional EMR of a grade 1 10-mm rectal NET with deep margin involvement ( Fig. 1 ). In order to achieve complete en bloc resection of the residual lesion with tumor-free margins, we performed intermuscular endoscopic dissection ( Video 1 ).

**Fig. 1:**
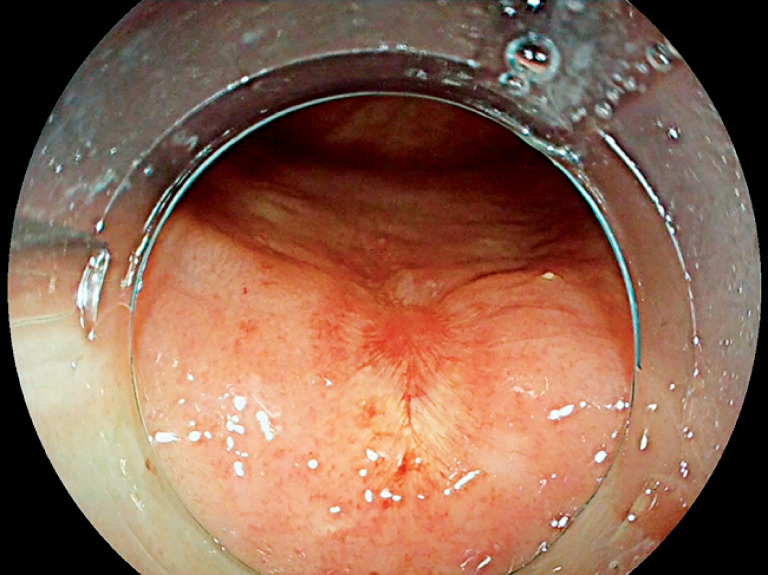
Residual rectal neuroendocrine tumor.

Endoscopic intermuscular dissection of a residual rectal neuroendocrine tumor with high-pressure injection and double-clip traction.Video 1

After marking the lesion, submucosal injection of glycerol mixed with indigo carmine and mucosal incision using a HybridKnife type I (Erbe Elektromedizin GmbH, Tübingen, Germany) were performed, followed by trimming of the submucosal layer. Then, a circumferential incision of the circular muscular layer was performed, with exposure of the longitudinal muscular layer ( Fig. 2 ). Exposure of the intermuscular plan was improved with double-clip countertraction, with the clip grasping the internal circular muscular layer ( Fig. 3 ). In addition, high-pressure glycerol injection through the HybridKnife was useful in dramatically enlarging the visible plan between the two muscular layers ( Fig. 4 ) and performing safe complete endoscopic resection without damage to the longitudinal muscular layer.

**Fig. 2:**
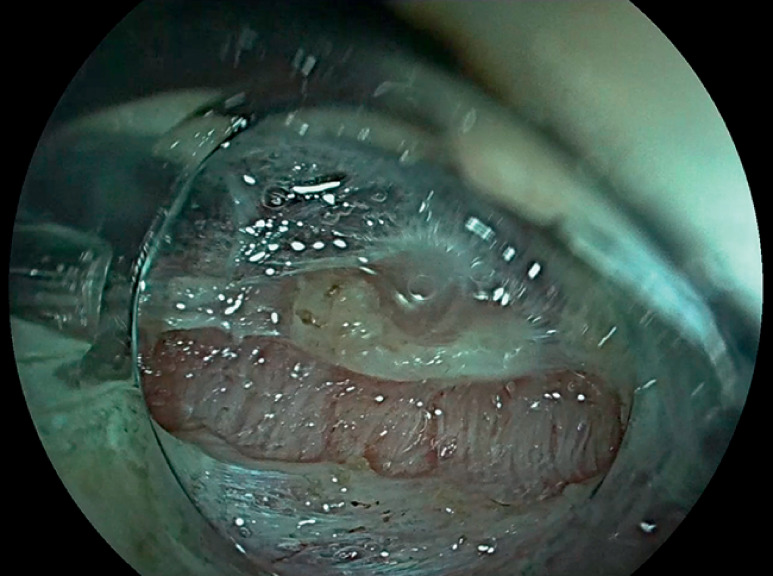
Incision of the circular muscular layer and visualization of the intermuscular space.

**Fig. 3:**
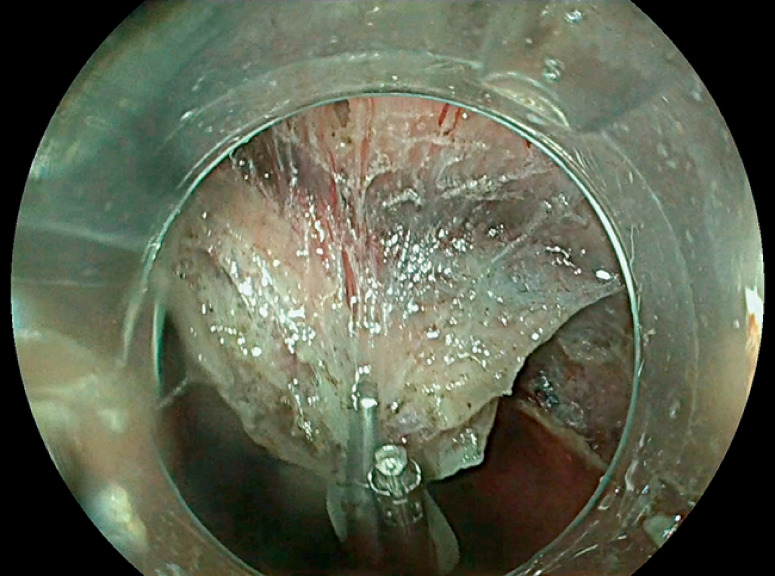
Double-clip traction increasing visualization of the intermuscular space.

**Fig. 4:**
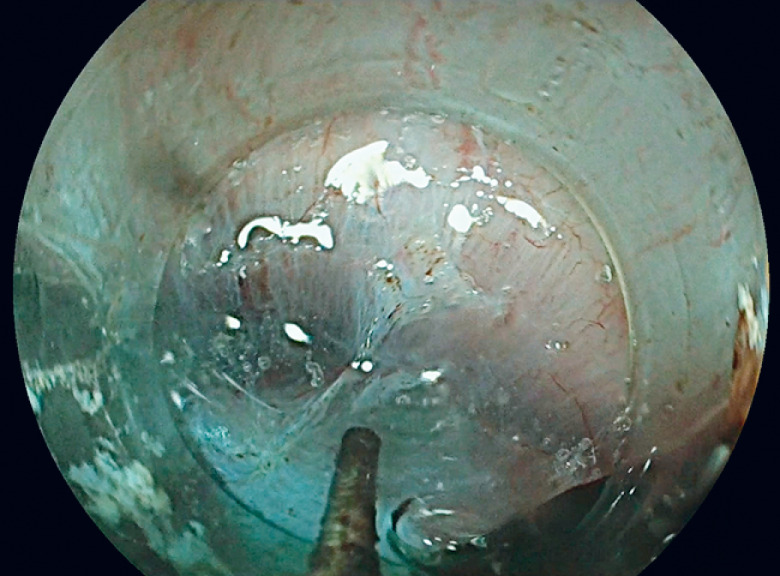
High-pressure intermuscular injection enlarging intermuscular space.

Histopathological examination of the specimen showed millimetric residual grade 1 well-differentiated NET, with lateral and deep free margins of more than 2 mm. The patient was discharged the same day and no adverse events occurred.

Endoscopic intermuscular dissection with traction and efficacious injection allows accurate resection of small rectal NETs, thus avoiding the risk of positive deep margins, and should be considered in order to protect against incomplete resection.

Endoscopy_UCTN_Code_TTT_1AQ_2AD

## Notes

Endoscopy E-Videos https://eref.thieme.de/e-videos
*E-Videos* is an open access online section of the journal *Endoscopy* , reporting on interesting cases and new techniques in gastroenterological endoscopy. All papers include a high-quality video and are published with a Creative Commons CC-BY license. Endoscopy E-Videos qualify for HINARI discounts and waivers and eligibility is automatically checked during the submission process. We grant 100% waivers to articles whose corresponding authors are based in Group A countries and 50% waivers to those who are based in Group B countries as classified by Research4Life (see: https://www.research4life.org/access/eligibility/ ). This section has its own submission website at https://mc.manuscriptcentral.com/e-videos .

## References

[1] Deprez PH Moons LMG OʼToole D Endoscopic management of subepithelial lesions including neuroendocrine neoplasms: European Society of Gastrointestinal Endoscopy (ESGE) Guideline Endoscopy 2022 54 412 429 10.1055/a-1751-5742 35180797

[2] Fine C Roquin G Terrebonne E Endoscopic management of 345 small rectal neuroendocrine tumours: a national study from the French group of endocrine tumours (GTE) United European Gastroenterol J 2019 7 1102 1112 10.1177/2050640619861883 PMC6794692 31662867

[3] de Mestier L Lepage C Baudin E Digestive neuroendocrine neoplasms (NEN): French Intergroup clinical practice guidelines for diagnosis, treatment and follow-up (SNFGE, GTE, RENATEN, TENPATH, FFCD, GERCOR, UNICANCER, SFCD, SFED, SFRO, SFR) Dig Liver Dis 2020 52 473 492 10.1016/j.dld.2020.02.011 32234416

[4] Moons LMG Bastiaansen BAJ Richir MC Endoscopic intermuscular dissection for deep submucosal invasive cancer in the rectum: a new endoscopic approach Endoscopy 2022 54 993 998 10.1055/a-1748-8573 35073588

